# A novel endoplasmic stress mediator, Kelch domain containing 7B (KLHDC7B), increased Harakiri (HRK) in the SubAB-induced apoptosis signaling pathway

**DOI:** 10.1038/s41420-021-00753-0

**Published:** 2021-11-19

**Authors:** Kinnosuke Yahiro, Kohei Ogura, Hiroyasu Tsutsuki, Sunao Iyoda, Makoto Ohnishi, Joel Moss

**Affiliations:** 1 Department of Microbiology and Infection Control Sciences, Division of Biological Sciences, Kyoto Pharmaceutical University, Kyoto, Japan; 2 Advanced Health Care Science Research Unit, Institute for Frontier Science Initiative, Kanazawa University, Kumamoto, Kumamoto Japan; 3 Department of Microbiology, Graduate School of Medical Sciences, Kumamoto University, Kumamoto, Kumamoto Japan; 4 Department of Bacteriology I, National Institute of Infectious Diseases, Tokyo, Japan; 5 Pulmonary Branch, National Heart, Lung, and Blood Institute, National Institutes of Health, Bethesda, MD USA

**Keywords:** Apoptosis, Stress signalling

## Abstract

Locus for Enterocyte Effacement (LEE)-positive Shiga-toxigenic *Escherichia coli* (STEC) contributes to many global foodborne diseases, with infection characterized by severe gastrointestinal symptoms, including bloody diarrhea. The incidence of LEE-negative STEC-mediated disease is also increasing globally. Subtilase cytotoxin (SubAB) is released by some LEE-negative STEC strains. It cleaves BiP, which is a chaperone protein located in the endoplasmic reticulum (ER), thereby causing apoptosis induced by ER stress. To date, the apoptotic signaling pathway mediated by SubAB has not been identified. In the current study, RNA-seq analysis showed that SubAB significantly induced the expression of Kelch domain containing 7B (KLHDC7B). We explored the role of KLHDC7B in the SubAB-induced apoptotic pathway. SubAB-induced KLHDC7B mRNA expression was increased after 12 h of incubation of toxin with HeLa cells. KLHDC7B expression was downregulated by knockdown of PKR-like endoplasmic reticulum kinase (PERK), CEBP homologous protein (CHOP), activating transcription factor 4 (ATF4), and CEBP β (CEBPB). KLHDC7B knockdown suppressed SubAB-stimulated CHOP expression, poly(ADP-ribose) polymerase (PARP) cleavage, and cytotoxicity. The over-expressed KLHDC7B was localized to the nucleus and cytosolic fractions. Next, we used RNA-seq to analyze the effect of KLHDC7B knockdown on apoptosis induced by SubAB, and found that the gene encoding for the pro-apoptotic Bcl-2 family protein, Harakiri (HRK), was upregulated in SubAB-treated control cells. However, this effect was not observed in SubAB-treated KLHDC7B-knockdown cells. Therefore, we identified the pathway through which SubAB-induced KLHDC7B regulates HRK expression, which is essential for apoptosis in toxin-mediated ER stress.

## Introduction

Serotype O157:H7 is the most common strain of Locus for Enterocyte Effacement (LEE)-positive, Shiga-toxigenic *Escherichia coli* (STEC). This serotype causes many food-borne diseases, which manifest as bloody diarrhea, hemorrhagic colitis, and hemolytic-uremic syndrome (HUS) [1]. Shiga toxin (Stx) 1 and/or 2 produced by most LEE-positive STEC are important virulence factors that trigger severe gastrointestinal diseases and HUS [2]. LEE-negative STEC strains may also cause infections, and the virulence of some of these strains results from the production of the cytotoxins, Stx2 and subtilase cytotoxin (SubAB) [3].

SubAB and Stx are cytotoxins that belong to the AB_5_ family [3], and are comprised of a catalytically active A subunit and a B subunit pentamer, which contains the receptor-binding domain [3]. SubAB, produced by LEE-negative STEC, recognizes sialic acid-modified glycoproteins on the human cell surface as receptors [4–6]. Binding to the receptor internalizes the toxin by endocytosis [7] and macropinocytic-like pathways [8]. SubAB is translocated from the Golgi to endoplasmic reticulum (ER) via pathways that involve the coatomer protein complex subunit Beta, sorting nexin, component oligomeric Golgi complex, Ras-related proteins [9], jumping translocation breakpoint protein, KDEL endoplasmic reticulum protein retention receptor 2 [6], and protein disulfide isomerase [10]. SubAB cleaves the chaperone protein BiP/Grp78 (Leu^416/417^) at a specific site [3], thereby activating proteins that detect ER stress, e.g., PERK, inositol-requiring enzyme (IRE1), and activating transcription factor 6 (ATF6) [11, 12]. These events are followed by caspase activation via mitochondrial cytochrome c release into the cytosol [13]. Furthermore, stress signaling inhibits protein synthesis [14], causes cell cycle arrest [14], decreases iNOS synthesis [15], promotes the formation of stress granules [16], and induces the production of a novel, non-secretory form of lipocalin 2 [17]. Additionally, when SubAB is administered intraperitoneally into the mice, severe fatal intestinal hemorrhage occur [18].

Kelch domain containing 7B (KLHDC7B) is a tumor marker with epigenetic differences in breast [19] and laryngeal [20] cancers. The promoter region of KLHDC7B is hyper-methylated in tumors compared to normal tissues [21]. KLHDC7B contains 594 amino acids, with a Kelch domain that, in generally, consists of five to seven repeated motifs [22]. KLHDC7B is a member of the Kelch family; the Kelch proteins play a role in various cellular events (e.g., cytoskeletal arrangement, cell morphology, protein degradation, gene expression) [23]. The cellular function of KLHDC7B is unclear.

Using RNA-seq analysis, we found that KLHDC7B mRNA was significantly increased in SubAB-treated cells 12 h after incubation. To determine the function and regulation of KLHDC7B in the SubAB-induced cell death signaling pathway, we analyzed RNA-seq data between control and KLHDC7B-knockdown cells with or without SubAB. SubAB-induced KLHDC7B was regulated by the PERK/ATF4/CEBPB/CHOP pathway. KLHDC7B was localized to the cytosolic fraction and nucleus. Harakiri (HRK) belongs to the Bcl-2 family and has pro-apoptotic functions. The level of HRK mRNA was significantly enhanced in SubAB-treated control cells, but not KLHDC7B-knockdown cells. Thus, HRK is a critical, pro-apoptotic protein controlled by SubAB-induced KLHDC7B. In this study, we identified the toxin-induced apoptosis pathway involved in the regulation of HRK expression (a critical cell death protein) by SubAB-stimulated KLHDC7B (a novel ER-stress death mediator).

## Results

### RNA-seq and differential expression analysis of SubAB-treated and control cells

**Fig. 1:**
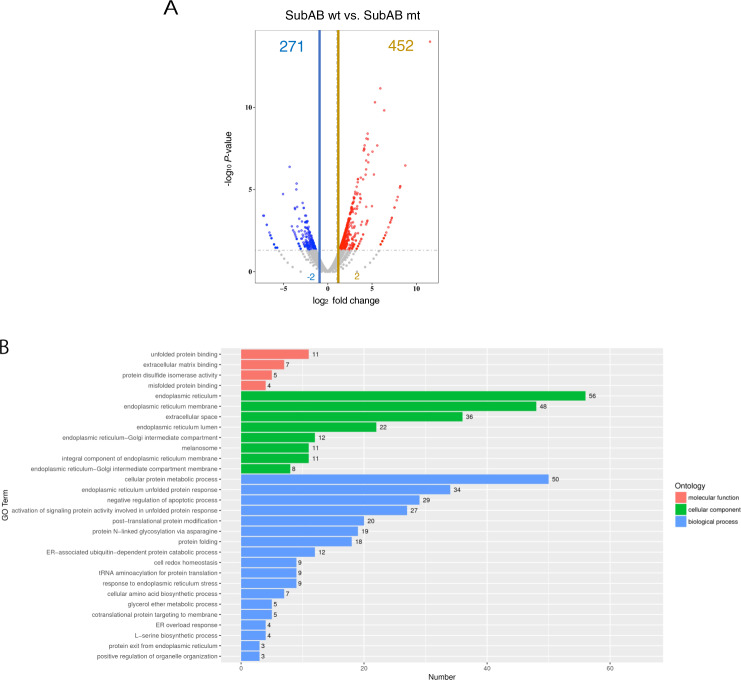
RNA-seq analysis of SubAB-treated HeLa cells. HeLa cells were incubated with 400 ng mL^−1^ of SubAB wild-type (wt) or inactivated mutant (mt) for 18 h, followed by RNA extraction. RNA-seq analysis was performed using GENEWIZ. **A** Volcano plot of differentially expressed genes (DEGs). DEGs were identified using the DESeq2 Bioconductor package, which is based on the negative binomial distribution. Dispersion and logarithmic fold change estimates included data-driven prior distributions; Padj of genes were set at <0.05 to identify DEGs. **B** Gene ontology (GO) term enrichment analysis of DEGs; increased GO terms in biological process, molecular function, and cellular component branches are displayed.

We used RNA-seq analysis to identify the novel cell death-related protein induced by SubAB in HeLa cells. The differentially expressed genes (DEGs) of SubAB-treated cells showed differences in levels of more than 2-fold with significant *p* values (<0.05). The gene expression patterns are depicted in the volcano plot analysis (Fig. 1A), along with the significantly increased (red dots, 452 genes) and decreased (blue dots, 271 genes) genes in SubAB-treated cells.

Gene Ontology (GO) analysis was used to determine the functional and biological processes active in SubAB-treated cells (Fig. 1B). The SubAB-enriched genes were participating in the biological processes of cellular protein metabolism and ER unfolded-protein response. Additionally, for the cellular components, genes involved in the ER or ER membrane were increased.

Table 1 summarizes the top 10 enriched mRNAs from SubAB-treated cells. SubAB-upregulated mRNAs (e.g., CHOP [13, 24], HSPA5 [25], CHAC1 [26], LCN2 [17], GDF15 [27], ASNS [28], HERPUD1 [29], ADM2 [30]) were affected by ER stress. KLHDC7B, a novel factor upregulated by ER stress, was significantly increased in response to wild-type but not mutant SubAB treatment.

**Table 1:** The top 10 mRNA enriched by SubAB are listed.

GeneSymbol	Description	Log2 ration
KLHDC7B	kelch domain containing 7B	11.531
ADM2	adrenomedullin 2	5.9341
SLC7A11	solute carrier family 7	5.3272
CHAC1	cation transport regulator homolog 1	6.37
DDIT3	DNA-damage-inducible transcript 3	4.4997
ASNS	asparagine synthetase (glutamine-hydrolyzing)	4.3619
GDF15	growth differentiation factor 15	4.524
HSPA5	heat shock 70 kDa protein 5	4.1605
LCN2	lipocalin 2	5.5862
HERPUD1	homocysteine-inducible endoplasmic reticulum stress-inducible ubiquitin-like domain member 1	4.1037

### KLHDC7B is involved in SubAB-induced apoptosis

**Fig. 2:**
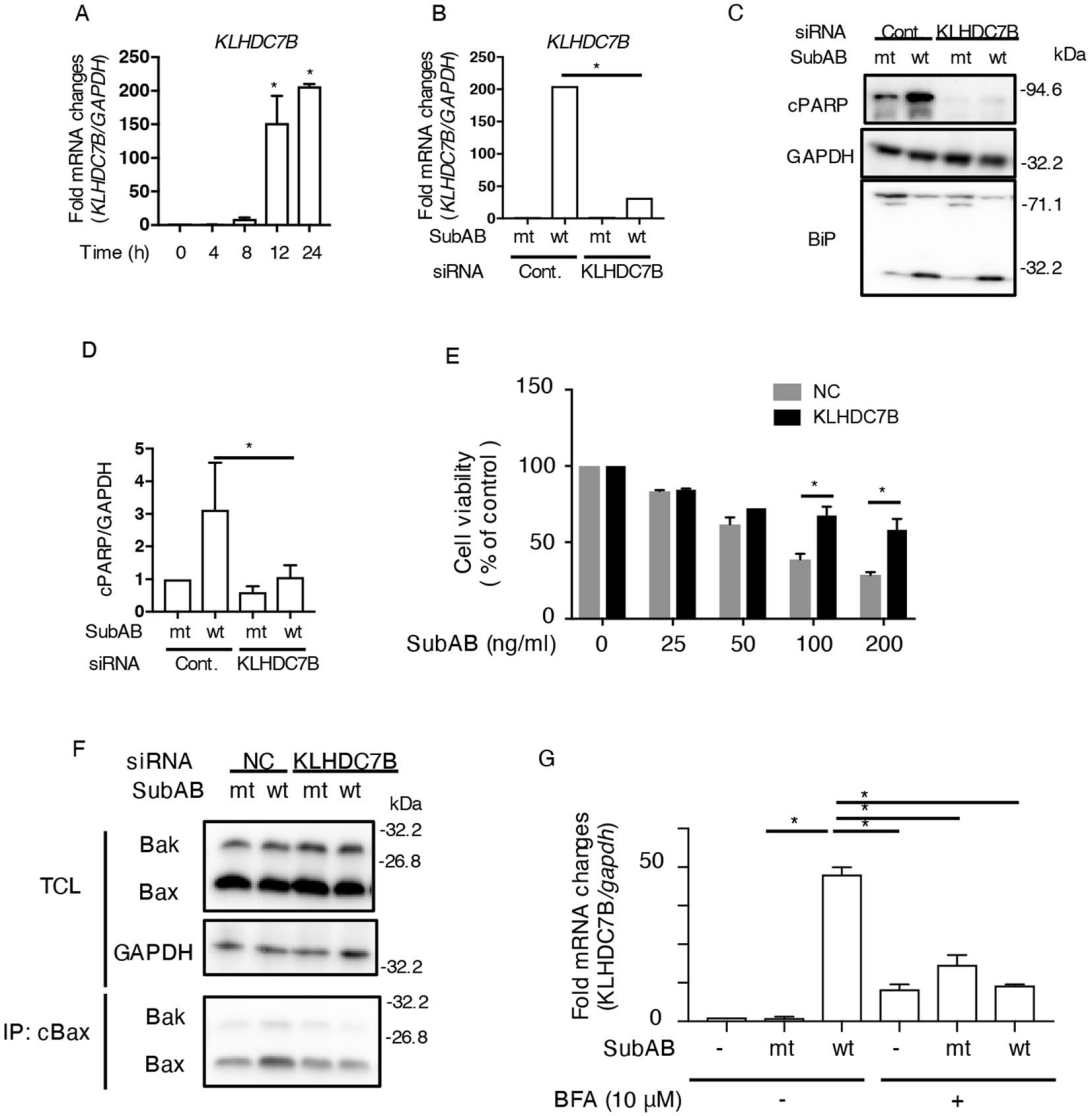
SubAB induces KLHDC7B expression. **A** HeLa cells were incubated with 400 ng mL^−1^ of SubAB for the indicated duration. The *KLHDC7B* mRNA levels were measured using RT-qPCR. GAPDH was used as the internal control. Data are presented as mean ± standard deviation from three independent experiments. *P < 0.05 versus untreated control cells. **B** Control (NC) or KLHDC7B siRNA-transfected HeLa cells were incubated for 24 h with 400 ng mL^−1^ of catalytically-inactive SubA_S272A_B (mt) or SubAB (wt). The mRNA levels of *KLHDC7B* were measured using RT-qPCR. GAPDH was used as the internal control. Data are presented as mean ± standard deviation (*n* = 3). *P < 0.05 versus mt SubAB-treated control cells. **C** Control (NC) or KLHDC7B siRNA-transfected cells were incubated for 16 h with 400 ng mL^−1^ of SubA_S272A_B (mt) or SubAB (wt). Immunoblotting with the antibodies was performed on cell lysates. GAPDH served as the loading control. **D** Densitometry was used to quantify the level of cPARP in HeLa cells. Data are presented as mean ± standard deviation from three independent experiments. *P < 0.05. **E** Control (NC) or KLHDC7B siRNA-transfected cells were incubated for 96 h with SubAB (wt). Cell viability was monitored using the Cell Counting Kit. Data are presented as mean ± standard deviation from three independent experiments. *P < 0.05. **F** The siRNA-transfected cells were incubated with mt or wt SubAB for 16 h; cells were lysed and immunoprecipitated using conformation-specific anti-Bax (cBax) monoclonal antibodies. SDS-PAGE was used to analyze the immunocomplexes (IP) or total cell lysates (TCL), followed by immunoblotting with anti-Bax and anti-Bak antibodies. Data are mean values from two or more independent experiments. **G** Cells were incubated with 10 μM of brefeldin A (BFA) with or without 400 ng mL^−1^ of SubA_S272A_B (mt) or SubAB (wt) for 16 h. The *KLHDC7B* mRNA levels were measured using RT-qPCR. GAPDH was used as the internal control. Data are presented as mean ± standard deviation from three independent experiments. *P < 0.05.

As shown by RNA-seq analysis, KLHDC7B mRNA was most significantly stimulated by SubAB (Table 1). First, we tested if SubAB treatment increases KLHDC7B mRNA level using real-time, quantitative PCR (RT-qPCR). The RNA-seq data set was used to design the primer sequences (Table 2). The expression level of KLHDC7B mRNA was significantly increased in HeLa cells after 12 h of incubation with SubAB (Fig. 2A) In addition, we also found that SubAB increased KLHDC7B mRNA levels in the human colon cancer HCT116 cell line (Fig. S1).

**Table 2:** List of primers used in this study.

Primer name	Sequences
FLAG-tagged KLHDC7B forward	5’-CAA GCT TGC GGC CGC CAC CATGATCCAGGGCACCTTG-3’
FLAG-tagged KLHDC7B reverse	5’-ACCGGATCCGTCGAC GAGTGAGGTCTGCAGCCGGT-3’
qPCR KLHDC7B forward	5’-GCACAACTACCTGTTTCTGGCG-3’
qPCR KLHDC7B reverse	5’-TGGCTCCAGATGTTGGTCAGAG-3’
qPCR CHOP forward	5’-GGTATGAGGACCTGCAAGAGGT-3’
qPCR CHOP reverse	5’-CTTGTGACCTCTGCTGGTTCTG-3’
qPCR GAPDH reverse	5’-GTCTCCTCTGACTTCAACAGCG-3’
qPCR GAPDH forward	5’-ACCACCCTGTTGCTGTAGCCAA-3’
qPCR CEBPB forward	5’-AGAAGACCGTGGACAAGCACAG-3’
qPCR CEBPB reverse	5’-CTCCAGGACCTTGTGCTGCGT-3’
qPCR CAPN5 forward	5’-AGTGTGAGGGAGACAAAGTCCG-3’
qPCR CAPN5 reverse	5’-CATCCTTCAGCACTCGGTGGTT-3’
qPCR GAPDH forward	5’-GTCTCCTCTGACTTCAACAGCG-3’
qPCR GAPDH reverse	5’-ACCACCCTGTTGCTGTAGCCAA-3’
qPCR HERPUD1 forward	5’-CCAATGTCTCAGGGACTTGCTTC-3’
qPCR HERPUD1 reverse	5’-CGATTAGAACCAGCAGGCTCCT-3’
qPCR CRELD2 forward	5’-AGAGACTGCGGCGAGTGTGAAG-3’
qPCR CRELD2 reverse	5’-TAGGAGCCGTTGGCGTTCTTAC-3’
qPCR NUPR1 forward	5’-GACTCCAGCCTGGATGAATCTG-3’
qPCR NUPR1 reverse	5’-CTTCTCTCTTGGTGCGACCTTTC-3’
qPCR GADD45A forward	5’-CTGGAGGAAGTGCTCAGCAAAG-3’
qPCR GADD45A reverse	5’-AGAGCCACATCTCTGTCGTCGT-3’
qPCR ATP8B2 forward	5’-CGGCTATTCCTGCAAGATGCTG-3’
qPCR ATP8B2 reverse	5’-GTCCTGATAGGTGAAGCCGTTG-3’
qPCR GADD34 forward	5’-TCCGACTGCAAAGGCGGCTCA-3’
qPCR GADD34 reverse	5’-CAGCCAGGAAATGGACAGTGAC-3’
qPCR PDIA4 forward	5’-CCAGCAGGTTTGATGTGAGTGG-3’
qPCR PDIA4 reverse	5’-GGAGACTTCTCTGACCTTGGCA-3’
qPCR HNF4A forward	5’-GGTGTCCATACGCATCCTTGAC-3’
qPCR HNF4A reverse	5’-AGCCGCTTGATCTTCCCTGGAT-3’
qPCR HRK forward	5’-AGGTTGGTGAAAACCCTGTG-3’
qPCR HRK reverse	5’-TTTCTACGATCGCTCCAGGC-3'

Next, we evaluated the effect of KLHDC7B knockdown by siRNA on SubAB-induced apoptosis. Control or KLHDC7B siRNA-transfected HeLa cells were incubated with toxins for 24 h. Then, we measured KLHDC7B mRNA levels by RT-qPCR. As shown in Fig. 2B, SubAB-stimulated KLHDC7B mRNA was significantly suppressed in KLHDC7B siRNA-transfected cells. Previous studies showed that SubAB-induced cell death was dependent on caspase activation-mediated apoptosis [12, 13]. In KLHDC7B-knockdown cells, SubAB-stimulated PARP cleavage, an apoptosis marker, was inhibited in comparison with the control siRNA-transfected cells, but not BiP cleavage (Fig. 2C, D). Next, we measured the effect of KLHDC7B knockdown on cell viability in the presence of SubAB. In control cells, SubAB decreased cell viability in a dose-dependent manner. Additionally, SubAB-induced cell death was attenuated in KLHDC7B-knockdown cells (Fig. 2E). As shown in Fig. 2C and E, SubAB-induced changes in the conformation of Bax/Bak were significantly suppressed in KLHDC7B-knockdown cells, in comparison with control siRNA-transfected cells (Fig. 2F).

Brefeldin A (BFA) inhibits vesicle formation and trafficking between the ER and Golgi apparatus [31], thereby inhibiting SubAB-mediated BiP cleavage in the ER [17]. To evaluate the effect of BFA on SubAB-increased KLHDC7B, cells were incubated with SubAB for 24 h with or without BFA. In the presence of BFA, SubAB-stimulated KLHDC7B mRNA was significantly suppressed; BFA alone increased KLHDC7B mRNA expression (Fig. 2G). Our results suggested that ER stress due to SubAB-induced BiP cleavage increased KLHDC7B.

### Localization of FLAG-tagged KLHDC7B in HeLa cells

**Fig. 3:**
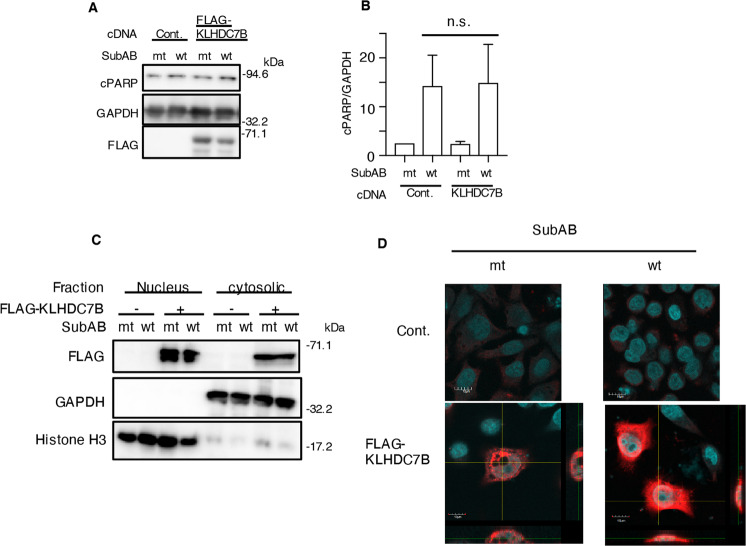
KLHDC7B is localized to the nuclear and cytoplasmic compartments. **A** Control or FLAG-tagged KLHDC7B plasmid-transfected cells were incubated for 8 h with 400 ng mL^−1^ of SubA_S272A_B (mt) or SubAB (wt). Immunoblotting was performed for the cell lysates. GAPDH served as the loading control. **B** Densitometry was used to quantify the cPARP levels in HeLa cells. Data are presented as mean ± standard deviation from three independent experiments. **C**, **D** Control or FLAG-tagged KLHDC7B plasmid-transfected cells were incubated for 16 h with 400 ng mL^−1^ of SubA_S272A_B (mt) or SubAB (wt). Cells underwent fractionation with the Nuclear/Cytosol Fractionation Kit (WAKO). Histone 3 and GAPDH served as the loading controls for the nuclear and cytosolic fraction, respectively (**C**). Localization of FLAG-tagged KLHDC7B was visualized by immunofluorescence stain with anti-FLAG antibodies. Data are representative of three independent experiments.

We evaluated the effect of overexpression of FLAG-tagged KLHDC7B on SubAB-induced apoptosis. In FLAG-tagged KLHDC7B-transfected cells, PARP cleavage did not increase in the presence of SubAB after 8 h of incubation (Fig. 3A, B). The localization of KLHDC7B has not been identified. To establish the subcellular distribution of KLHDC7B, we performed cell fractionation experiments using FLAG-tagged KLHDC7B-transfected HeLa cells, and investigated whether the subcellular localization of KLHDC7B changed in response to SubAB. We found that KLHDC7B localized to both the cytoplasmic and nuclear compartments, despite adding SubAB (Fig. 3C). To confirm the fractionation results, we stained FLAG-tagged KLHDC7B with immunofluorescence-labeled antibodies and used the DNA-binding fluorochrome DAPI. Consistent with the fractionation results, FLAG-tagged KLHDC7B (red) was localized to both the cytosolic and nuclear compartments, suggesting that the localization of FLAG-tagged KLHDC7B was not affected by SubAB (Fig. 3D).

### SubAB-induced activation of the ATF4/CHOP/CEBPB pathway regulates KLHDC7B expression

**Fig. 4:**
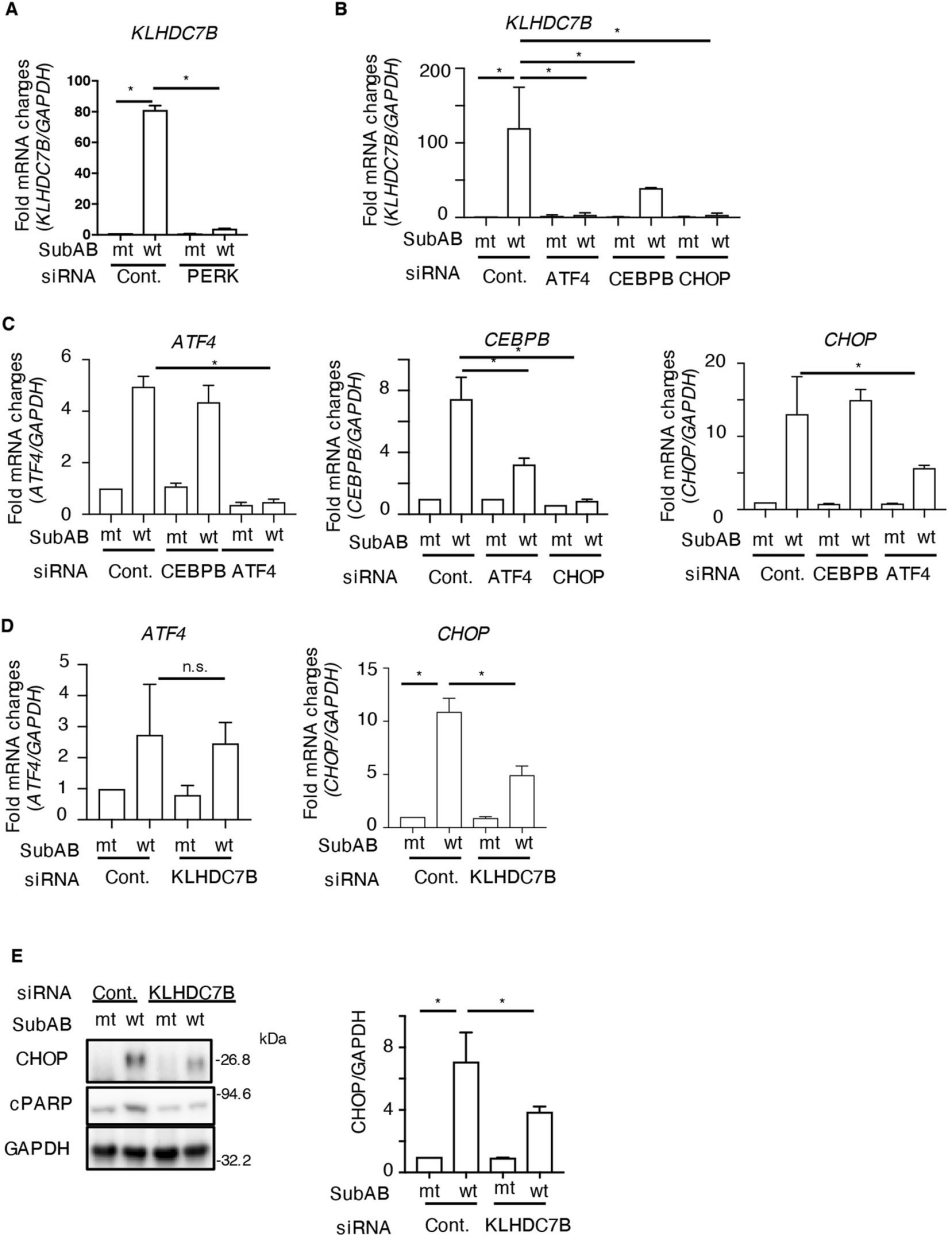
KLHDC7B was regulated by PERK/ATF4/CEBPB. **A**–**D** siRNA-transfected cells were incubated for 16 h with 400 ng mL^−1^ of SubA_S272A_B (mt) or SubAB (wt). The *KLHDC7B, ATF4, CEBPB, and CHOP* mRNA levels were measured using RT-qPCR. GAPDH was used as the internal control. Data are presented as mean ± standard deviation (*n* = 3). *p < 0.05. **E** Control (NC) or KLHDC7B siRNA-transfected cells were incubated for 16 h with 400 ng mL^−1^ of SubA_S272A_B (mt) or SubAB (wt). Immunoblotting was performed on the cell lysates and GAPDH served as the loading control. Densitometry was used to quantify the CHOP level in HeLa cells. Data are presented as mean ± standard deviation from three independent experiments. *p < 0.05.

Previous studies demonstrated that SubAB-stimulated ER stress activates ER stress sensor proteins, which subsequently activate transcription factors and induce several proteins [10–12,16, 18, 32, 33]. We attempted to identify the pathway that regulates SubAB-stimulated KLHDC7B expression. PERK is an essential ER stress sensor that plays a role in apoptosis induced by SubAB [12]. SubAB-induced increase in KLHDC7B mRNA expression level was significantly attenuated in PERK-knockdown cells (Fig. 4A). We examined the effect of PERK-regulated transcription factors (e.g., ATF4, CHOP, and CEBPB) on KLHDC7B expression. As shown in Fig. 4B, knockdown of ATF4 and CHOP by siRNA significantly inhibited SubAB-stimulated KLHDC7B expression, which was partially suppressed in CEBPB-knockdown cells.

Next, we determined the order of ATF4, CHOP, and CEBPB in SubAB-stimulated KLHDC7B expression. In ATF4-knockdown cells, SubAB-induced increase in CHOP and CEBPB mRNA levels were attenuated. Although SubAB-induced increase in CHOP mRNA expression level was not attenuated in CEBPB-knockdown cells, knockdown of CHOP suppressed SubAB-induced increase in CEBPB mRNA expression levels (Fig. 4C). We further investigated whether KLHDC7B knockdown affects SubAB-induced activation of transcription factors, e.g., CHOP, ATF4. In KLHDC7B-knockdown cells, SubAB-induced increase in ATF4 mRNA expression level was not changed, but CHOP mRNA expression level was significantly decreased (Fig. 4D). Consistent with the RT-qPCR results, the Western blotting analysis showed that SubAB-induced increase in CHOP expression was attenuated in KLHDC7B-knockdown cells (Fig. 4E), suggesting that SubAB-induced KLHDC7B expression is regulated by the PERK/ATF4/CHOP/CEBPB signaling pathway, and that the expressed KLHDC7B controls CHOP.

### Identification of KLHDC7B-regulating factors in SubAB-treated cells by RNA-seq and differential expression analysis

**Fig. 5:**
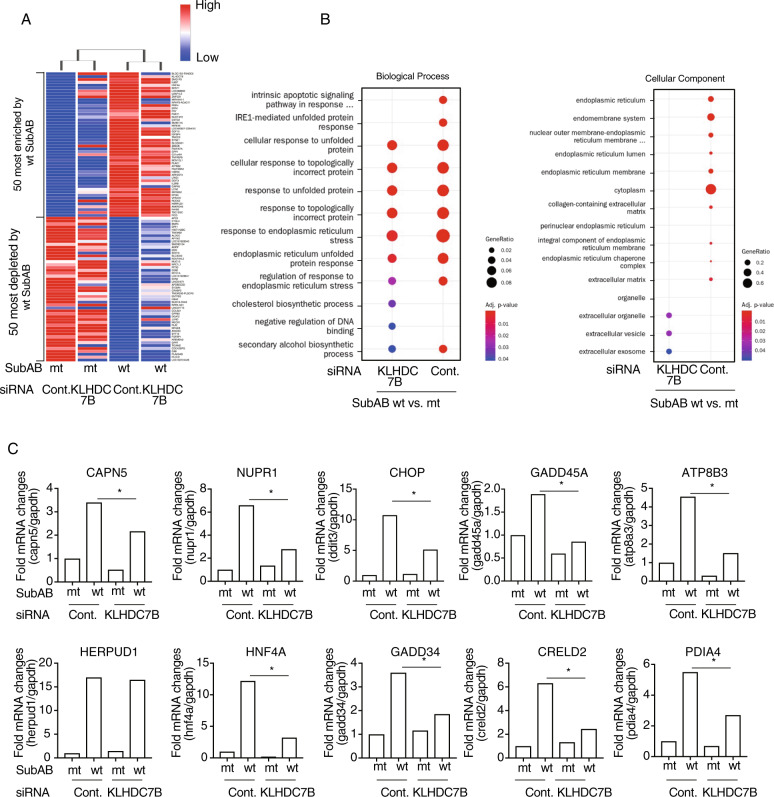
RNA-seq analysis between control and KLHDC7B knockdown cells. Control or KLHDC7B siRNA-transfected cells were incubated for 16 h with 400 ng mL^−1^ of SubA_S272A_B (mt) or SubAB (wt), and then purified mRNA. These mRNAs were investigated by RNA-seq analysis. **A** Heat map of most enriched or depleted 50 differentially expressed genes. **B** The RNA-seq results are summarized in two categories: biological process and cellular components. **C** The mRNA levels were measured using RT-qPCR. GAPDH served as the internal control and *CHOP* as the positive control. Data are representative of two independent experiments. *p < 0.05.

The function of KLHDC7B remains unknown. To explore the role of SubAB-stimulated KLHDC7B, we performed RNA-seq analysis using control or KLHDC7B siRNA-transfected cells with mutant or wild-type SubAB. A total of 100 million paired end reads, with an average of 50 million reads per sample, were mapped on a reference human genome UCSC GRCh37/hg19 (Table 3). A heat map analysis indicated that wild-type SubAB treatment resulted in different gene expression (enriched or depleted changes of host transcripts) between the control and KLHDC7B siRNA-transfected cells (Fig. 5A). The analysis of biological processes and cellular components suggested SubAB increased (i) intrinsic apoptotic signaling pathway, (ii) IRE1-mediated, unfolded-protein response, and (iii) regulation of response to ER stress, which were significantly suppressed in KLHDC7B-knockdown cells (Fig. 5B). Similar to the results from the RNA-seq data, RT-qPCR analysis showed that SubAB-induced increase in the mRNA levels (e.g., *CAPN5, NUPR1, CHOP, GADD45A, ATP8B3, HERPUD1, HNF4A, GADD34, CRELD2, PDIA4*) were significantly attenuated in KLHDC7B-knockdown cells (Fig. 5D).

**Table 3:** Mapped data stats of RNA-seq analysis.

Library	Total reads	Read length (bp)	Mapped reads	Mapped reads (%)
Cont._mt SubAB	53,417,956	101	52,299,104	97.91
Cont._wt SubAB	51,471,614	101	50,456,121	98.03
KLHDC7B siRNA_mt SubAB	55,435,614	101	54,409,690	98.15
KLHDC7B siRNA_wt SubAB	49,729,780	101	48,569,520	97.67

### KLHDC7B regulates the expression of HRK, which is essential for SubAB-induced apoptosis

**Fig. 6:**
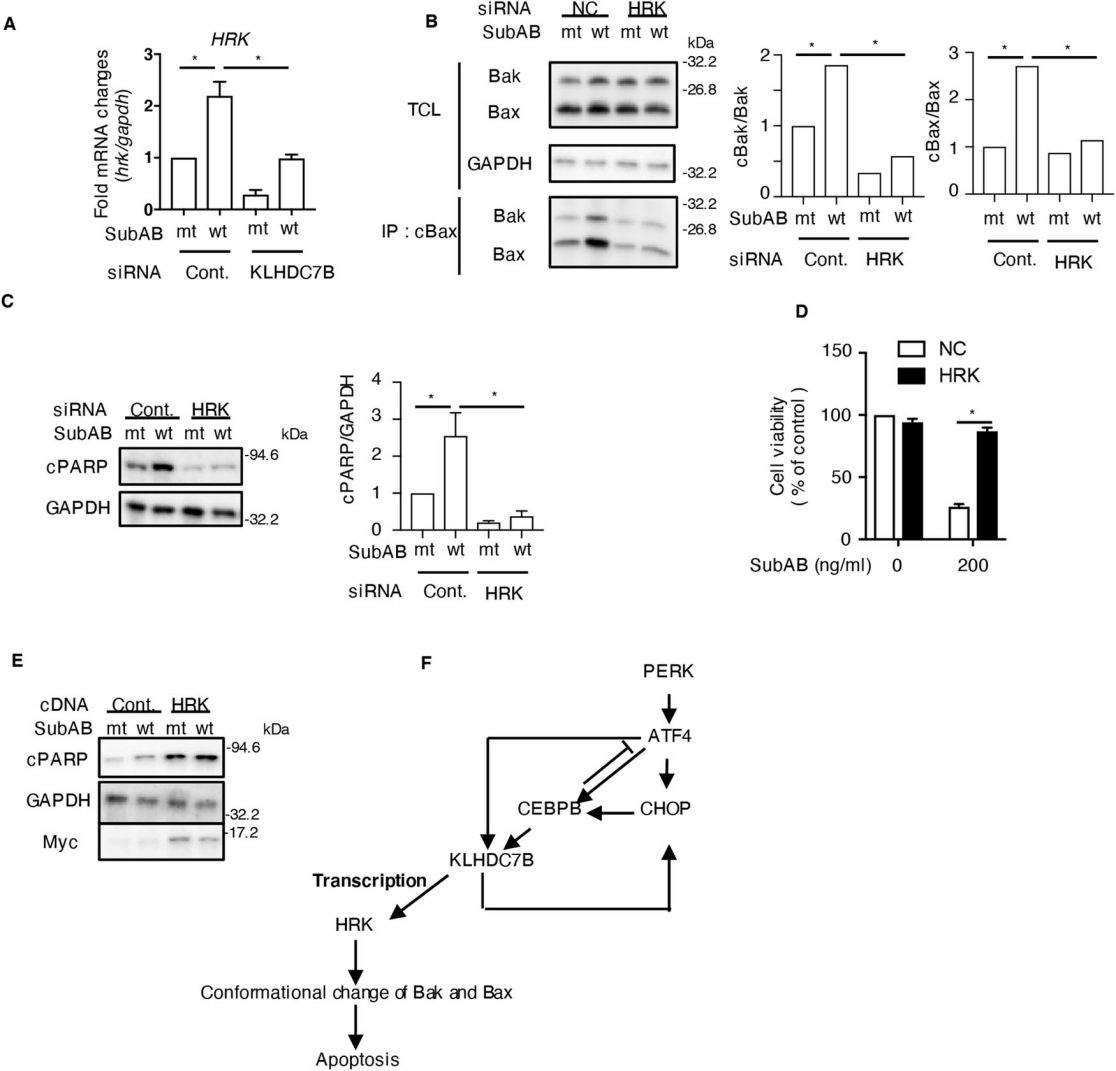
RNA-seq analysis of control and KLHDC7B knockdown cells. **A** The siRNA-transfected cells were incubated for 20–24 h with mt or wt SubAB. Then, the *HRK* mRNA levels were measured using RT-qPCR. GAPDH was used as the internal control. Data are presented as mean ± standard deviation (*n* = 3). *p < 0.05. **B** CHAPS buffer extracts from the siRNA-transfected cells were treated with mt or wt SubAB, and proteins were co-immunoprecipitated with conformation-specific, anti-active Bax monoclonal antibody (cBax). SDS-PAGE was used to analyze the immunocomplexes and total cell lysates (TCL), followed by immunoblotting with antibodies. A representative blot of two independent experiments is shown. Conformationally-changed Bax and co-precipitated Bak were quantified using a densitometer (right panel). **C** The siRNA-transfected cells were incubated for 20–24 h with mt or wt SubAB. Then, the cell lysates underwent immunoblotting with the indicated antibodies. GAPDH served as the loading control. Densitometry was used to quantify the cPARP level in HeLa cells. Data are presented as mean ± standard deviation from three independent experiments. *p < 0.05. **D** siRNA-transfected cells were incubated for 96 h with mt or wt SubAB, and observed for cell viability using the Cell Counting Kit. Data are presented as mean ± standard deviation from three independent experiments. *p < 0.05. **E** The cDNA-transfected cells were incubated for 16 h with mt or wt SubAB. Then, the cell lysates underwent immunoblotting with the antibodies. GAPDH served as the loading control. Densitometry was used to quantify the cPARP level in HeLa cells. Data are presented as mean ± standard deviation from three independent experiments. *p < 0.05. **F** The proposed model of SubAB-increased KLHDC7B regulates apoptotic cell death pathway via HRK expression.

To investigate how SubAB-induced increase in KLHDC7B regulates apoptosis, we searched for the apoptosis-related genes that were increased or decreased by SubAB in control cells, and showed an opposite response in KLHDC7B-knockdown cells. In RNA-seq analysis, we found that a gene encoding for the pro-apoptotic protein HRK was significantly increased by SubAB in control cells, but decreased by SubAB in KLHDC7B-knockdown cells. Using RT-qPCR analysis, in control cells, SubAB increased *HRK* mRNA levels, whereas decreased it in KLHDC7B-knockdown cells (Fig. 6A). We found that, similar to SubAB, a general ER stress inducer, tunicamycin (TM), also increased mRNA levels of CHOP, KLHDC7B, and HRK expression (Fig. S2). Although we examined the expression level of HRK by Western blotting using commercial anti-HRK antibodies (i.e., #NBP1-59447, Novus Biologicals; #PRS3771, Sigma Aldrich), we did not detect a specific human HRK band. HRK interacts with Bcl-2 and Bcl-xL, which are members of the anti-apoptotic Bcl protein family that inhibits HRK-induced cell death [34]. Previous studies showed that SubAB triggered changes in the conformation of Bax and Bak, which form a complex on the mitochondria [12, 13, 18, 35]. Next, we investigated the effect of HRK knockdown on conformational changes in Bax/Bak induced by SubAB using immunoprecipitation. The *HRK* mRNA level was significantly lower by HRK siRNA, which suppressed SubAB-induced increase in HRK mRNA expression levels (Fig. S3). Furthermore, SubAB-induced conformational changes in Bax/Bak were attenuated in HRK-knockdown cells (Fig. 6B). Consistent with these results, SubAB-induced PARP cleavage was significantly suppressed in HRK-knockdown compared to control cells (Fig. 6C). Further, in control cells, SubAB decreased cell viability after 96 h of incubation, while SubAB-induced decreased cell viability was inhibited in the HRK-knockdown cells (Fig. 6D). In cells overexpressing HRK, SubAB-induced PARP cleavage was enhanced (Fig. 6E). These data showed that increased HRK expression alone enhanced PARP cleavage, and this effect was enhanced with SubAB. Our findings suggest that SubAB-induced apoptosis is dependent on the mitochondria, and involves KLHDC7B-mediated HRK expression, followed by Bak/Bax-conformational changes.

## Discussion

To elucidate the mechanism of SubAB-induced apoptotic signaling, we performed RNA-seq analysis. Compared with inactivated SubAB mt, SubAB wt induced significant upregulation of 452 genes and downregulation of 271 genes (Fig. 1A). As shown in the GO term enrichment analysis (Fig. 1B), SubAB-induced cleavage of BiP stimulated multiple ER responses, including upregulation of ER stress markers, e.g., ATF4, CHOP genes (Fig. 4C). A recent study in HeLa cells reported that chemical ER stress inducers stimulated ATF4, followed by induction of mRNA expression of the inflammasome component, NLR Family Pyrin Domain Containing 1 (NLRP1), in HeLa cells [36]. The study demonstrated that the IRE1 and PERK pathways were important for the induction of NLRP1 mRNA expression and that ATF4, but not XBP-1, was critical for the induction. They also suggested that factors other than XBP-1 were involved in the IRE1 pathway. In our RNA-seq results, we observed that the inflammasome-related genes were not increased in SubAB-treated HeLa cells. These findings suggest that the contribution of different activated pathways may differ between chemical ER stress inducers and SubAB.

Using siRNA-knockdown cells, we found that KLHDC7B is a novel cell-death mediator. Although the function of KLHDC7B remains unknown, increased expression of KLHDC7B was observed in various tumors. However, the expression level was not always higher than that observed in non-tumor tissues [19]. In human breast cancer MCF cells, proliferation was decreased in KLHDC7B-overexpressing cells, and increased in KLHDC7B-knockdown cells [37]. In contrast, in the current study, we demonstrated that SubAB-induced apoptosis was significantly decreased in KLHDC7B-knockdown HeLa cells, suggesting that KLHDC7B was associated with cell proliferation, but the role of KLHDC7B may differ, depending on the tissue or stimulator.

SubAB-induced ER stress stimulated dramatic upregulation of KLHDC7B mRNA expression. The promoter region of KLHDC7B is a rarely hypermethylated gene in breast cancer cell lines [21]. We investigated whether the hypermethylation of the KLHDC7B promoter in HeLa cells participated in the expression by SubAB-induced ER stress. DNA methyltransferase inhibitor RG108 and 5-Aza-2’-deoxycytidine (5-Aza) did not affect SubAB-increased KLHDC7B mRNA expression levels or SubAB-induced PARP cleavage (Fig. S4A and B), suggesting that promoter methylation was not involved in KLHDC7B expression in HeLa cells. Our results suggest that SubAB-stimulated KLHDC7B mRNA expression level was regulated by increased ATF4 directly or indirectly by a pathway involved in CHOP or CEBPB. Interestingly, KLHDC7B knockdown suppressed SubAB-increased CHOP expression, suggesting that KLHDC7B is related to CHOP expression.

SubAB-induced apoptosis was mediated by conformational changes and effects of oligomerization of Bax/Bak on mitochondria, cytochrome *c* release, and caspase activation [13]. The pathway was suppressed by the anti-apoptotic Bcl-2 family member Bcl-xL- overexpressing cells [18]. These findings were consistent with the observation that Bcl-xL blocks the physical encounter of Bax and Bak on mitochondria, which is necessary for subsequent apoptosis. However, the molecular mechanisms leading to Bax/Bak conformational changes remain poorly defined. Besides Bcl-xL, previous studies showed that several factors, including Bid translocation from the cytosol to mitochondria [38], Bif-1 [39], apoptosis-associated speck-like protein (ASC) [40], Ku70 [41], 14-3-3 theta [42], and p53 [43], have participated in the regulation of Bax/Bax activity. Here, we identified by RNA-seq analysis data that SubAB induced an increase in HRK, regulated by KLHDC7B, involved in the generation of conformationally different Bax/Bak, followed by apoptosis. HRK has a BH3 region of the Bcl-2 member and controls apoptosis by inhibiting the pathway with anti-apoptotic Bcl-2 and Bcl-xL [34]. Previous studies reported that HRK participates in cell death, which is suppressed in Bcl-2- and/or Bcl-xL-overexpressing cells [44, 45]. Knockdown of HRK expression by siRNA significantly suppressed SubAB-induced apoptotic pathway and increased cell viability, suggesting that HRK is an essential apoptotic factor. These findings suggest that SubAB-induced increase in HRK inhibited Bcl-xL and/or Bcl-2 activity, followed by promotion of Bax/Bak conformational changes, resulting in cell death.

Previous reports showed that HRK mRNA in human tissues was highly expressed in spleen and bone marrow, and expressed at low levels in kidney, liver, lung, and brain [34]. Analysis of the HRK promoter region showed that *HRK* transcription was controlled by c-Jun, ATF2 [46, 47], and transcription repressor DREAM [48]. A recent study showed that Yes-associated protein (YAP)-knockdown increased HRK expression in neuroblastoma, suggesting that YAP is a suppressor of HRK expression [49]. AT-rich interactive domain-containing protein 1 A (ARID1A) is a subunit of the SWI/SNF chromatin remodeling complexes which bind DNA. Knockdown of ARID1A decreased HRK expression without direct binding to the *HRK* gene in gastric cancer cells [50]. Therefore, HRK expression is regulated by various transcription factors. KLHDC7B knockdown inhibited the expression of *HRK* transcription by SubAB. Although we could not detect a direct interaction between KLHDC7B and HRK promoter region by a ChIP assay, KLHDC7B might be a novel regulator as a transcription factor of HRK expression.

Figure 6F summarizes the proposed model of SubAB-increased KLHDC7B mediated cell death pathway. SubAB-activated PERK increased expression levels of ATF4 and CHOP, followed by upregulation of CEBPB. These transcription factors induced KLHDC7B expression, which upregulated HRK expression. HRK caused conformational changes of Bak/Bax, thereby causing cell death.

## Materials and methods

### Preparation of subtilase cytotoxin

Recombinant, His-tagged, wild-type subtilase cytotoxin (wt SubAB) or catalytically-inactivated mutant SubA (S272A)B (mt SubAB) was prepared in *Escherichia coli (E. coli)* BL21, followed by purification with Ni-nitrilotriacetic acid (NTA) agarose (Qiagen, Hilden, Germany), similar to the previously published procedure [14].

### Antibodies and other reagents

Antibodies targeting cleaved PARP (cPARP) (#5625), CHOP/GADD153 (#2895), PERK (#3192), Bax (#2772), and Bak (#3814) were purchased from Cell Signaling Technology (Beverly, MA, USA). Mouse monoclonal antibodies against BiP/GRP78 (#610978) and Bax (#610982) were purchased from BD Biosciences (Franklin Lakes, NJ, USA). Anti-FLAG (#014-22383) monoclonal antibody was purchased from Fujifilm WAKO Pure Chemical Corp (Tokyo, Japan). Anti-FLAG (#66008-3-Ig) and anti-GAPDH antibodies (#10494-1-AP) were purchased from Proteintech (Wuhan, China). Myc-DDK-tagged HRK expression plasmid was purchased from OriGene (Rockville, MD, USA).

### Purification of RNA for RNA-seq

HeLa cells were cultured in 6-well plates for 16 h with mt or wt SubAB (400 ng mL^−1^). Total RNA was purified using ISOGEN II (Nippon Gene Co., Ltd., Toyama, Japan). The paired end RNA-seq libraries were constructed using TruSeq Strand mRNA LT Sample Prep Kit and sequenced using Illumine HiSeq 2500. Therefore, 101 pair length reads were obtained using Genewitz Inc. (South Plainfield, NJ, USA) or Macrogen Japan (Kyoto, Japan).

### Differentially expressed genes and functional enrichment

Read counts and normalization values are determined by the transcript length and depth of coverage, and therefore indicate gene expression profiles. Fragments per kilobase of transcript per million mapped reads (FPKM) values or reads per kilobase of transcript per million mapped reads (RPKM) are often used as normalization values. The gene expression levels were compared between controls and toxin-treated cells to identify significant differentially expressed genes (DEGs). Among the DEGs, we selected those that had a greater than 2-fold difference and *p*-value < 0.05 compared to control cells. In addition, these DEGs were used for functional enrichment analysis using Gene Ontology (GO) and Kyoto Encyclopedia of Genes and Genomics (KEGG) pathway.

### Cell culture and gene transfection

HeLa cells (RIKEN, RCB0007) were cultured in RPMI1640 medium (Sigma-Aldrich, St. Louis, MO, USA). The medium contained 10% fetal calf serum and penicillin-streptomycin solution (Sigma-Aldrich). The cells were plated with a medium containing 10% FCS. RNA interference-mediated gene knockdown was performed using validated Qiagen HP small-interfering RNAs (siRNAs) for PERK (SI02223718) and C/EBPB (SI02777292). ATF4 siRNA was designed and validated, as described previously [51]. CHOP and KLHDC7B siRNAs were purchased from Santa Cruz Biotechnology (Santa Cruz, CA, USA). HRK siRNA (s194952) was purchased from Ambion (Austin, TX, USA). Cells were transfected with 50–100 nM of siRNAs for 48 h using Lipofectamine^TM^ RNAiMax transfection reagent (Invitrogen, Carlsbad, CA, USA), as per the manufacturer’s instructions. Protein knockdown was confirmed by RT-qPCR or immunoblotting with the aforementioned antibodies.

### Construction of FLAG-tagged KLHDC7B

ISOGEN II was used to purify the total RNA from SubAB-treated HeLa cells. Then, PrimeSTAR HS DNA Polymerase kit (TaKaRa Bio Inc., Shiga, Japan) was used to amplify cDNA in a 25-μL PCR mixture, as per the manufacturer’s instructions. The PCR conditions for KLHDC7B included 30 cycles of 98 °C for 15 s, 60 °C for 10 s, and 72 °C for 3 min. Table 2 summarizes the primers used for PCR. The PCR products were ligated into the Not1 and Sal1 sites of FLAG-5a expression vector (Sigma-Aldrich) using In-Fusion HD Cloning Kit (TaKaRa Bio Inc.). The FLAG-tagged human KLHDC7B plasmid was sequenced and transfected into HeLa cells using Polyethylenimine Max (Polysciences Inc. Warrington, PA, USA) in OPTI-MEM I reduced-serum medium (ThermoFisher Scientific, Waltham, MA, USA).

### Real-time qPCR

PrimeScript™ RT Master Mix (TaKaRa Bio Inc.) was used to amplify the cDNA in 10 μL of PCR mixture, as per the manufacturer’s instructions. KOD SYBER qPCR Mix (Toyobo, Osaka, Japan) and ABI Prism 7000 (PerkinElmer Life Sciences, Boston, MA, USA) were used to perform RT-qPCR. Table 2 shows the primers used for PCR. Relative expression levels were normalized to GAPDH and calibrated to the respective controls.

### Immunoblot analysis

Cell proteins were separated using SDS-PAGE and transferred to PVDF membranes, followed by incubation with the aforementioned primary antibodies. Horseradish peroxidase-labeled goat anti-mouse or anti-rabbit secondary antibodies (R&D systems, Abingdon, UK) were used for detection, followed by enhanced chemiluminescence (EzWestLumi One; ATTO Corp., Tokyo, Japan). Las 1000 (FUJIFILM) was used to visualize the bands and the Image Gauge software (FUJIFILM) was used for densitometric analysis of the scanned blots. The protein levels were normalized to GAPDH.

### Immunoprecipitation of conformationally-changed Bak/Bax

The siRNA-transfected cells (3 × 10^5^ cells/12-well plate) were incubated with 400 ng/mL of SubAB or mt SubAB for 12 h at 37 °C. Co-immunoprecipitation of conformationally-changed Bax or Bak followed the methods described in previous studies [12].

### Immunofluorescence confocal microscopy

Immunofluorescence analysis was performed as described previously [8, 12]. Briefly, FLAG-tagged KLHDC7B-transfected HeLa cells (3 × 10^5^ cells/12-well plate) on glass (Matsunami Glass Co. Ltd., Osaka, Japan) were incubated with 400 ng mL^−1^ of SubAB or mt SubAB. Cells were fixed with 4% paraformaldehyde (PFA), rinsed three times with PBS, and incubated with the blocking buffer (5% goat serum and 0.3% Triton X-100 in PBS) at room temperature for 1 h. Cells were further incubated with anti-FLAG antibodies (#014-22383, WAKO) in 0.4% BSA/PBS buffer at 4 °C overnight, washed twice with PBS, and incubated with anti-mouse Cy3 (#ab97035, Abcam, Cambridge, UK) antibodies at room temperature for 1 h in the dark.

After the cells were washed with PBS, they were mounted on glass slides using Prolong Gold Antifade reagent with DAPI (ThermoFisher Scientific). FV10i-LIV confocal microscopy (Olympus, Tokyo, Japan) was used to visualize the stained cells and the images were arranged with Adobe Photoshop CS4.

### Statistics

Student’s *t*-test was used for the densitometric analysis and analysis of RT-qPCR assays using the Graphpad Prism software (Graphpad Inc., San Diego, CA, USA). *P*-values < 0.05 were considered to indicate statistical significance.

## Supplementary information

Supplemental Figure legend

Fig.S1 HCT116 cells were incubated with 400 ng mL of SubAB for 18 h. The *KLHC7B* mRNA levels were measured using RT-qPCR. GAPDH served as the internal control. Data are presented as mean± standard deviation (n = 3). *p < 0.05.

Fig.S2 HeLa cells were incubated with 1 μg mL of Tunicamycin (TM) for 16 h. The KLHDC7B mRNA levels were measured using RT-qPCR. GAPDH served as the internal control. Data are presented as mean ± standard deviation (n = 3). *p < 0.05 versus untreated control cells.

Fig.S3 The siRNA-transfected cells were incubated for 48–72 h, followed by incubation with 400 ng mL of mt or wt SubAB for 18 h. The HRK mRNA levels were measured using RT-qPCR. GAPDH served as the internal control. Data are presented as mean ± standard deviation (n = 3). *p < 0.05.

Fig.S4 A, HeLa cells were incubated with 400 ng mL of SubAB with or without 200 μM of RG108 or 5 mM of 5’-AZA for 18–24h. The *KLHDC7B* mRNA levels were measured using RT-qPCR. GAPDH served as the internal control. B, After the cells were treated using the aforementioned method, cell lysates underwent immunoblotting with antibodies. GAPDH served as the loading control. Densitometry was used to quantify the cPARP level in HeLa cells. Similar results were obtained from two independent experiments.

## Data Availability

The raw reads data of RNAseq in this study are available in DNA Data Bank of Japan (DDBJ) under the accession number DRA012635.
