# Supplementary material for: A novel endoplasmic stress mediator, Kelch domain containing 7B (KLHDC7B), increased Harakiri (HRK) in the SubAB-induced apoptosis signaling pathway
Source: Cell Death Discov. 2021 Nov 19;7:360. doi: 10.1038/s41420-021-00753-0 (PMC8605022; doi:10.1038/s41420-021-00753-0)
Supplement: Supplementary file 1 — Supplemental Figure legend [file 41420_2021_753_MOESM1_ESM.docx]

**Supplemental Figure legends**

Figure S1.

HCT116 cells were incubated with 400 ng mL^-1^ of SubAB for 18 h. The *KLHDC7B* mRNA levels were measured using RT-qPCR. GAPDH served as the internal control. Data are presented as mean ± standard deviation (n = 3). **p* < 0.05.

Figure S2.

HeLa cells were incubated with 1 μg mL^-1^ of Tunicamycin (TM) for 16 h. The *KLHDC7B* mRNA levels were measured using RT-qPCR. GAPDH served as the internal control. Data are presented as mean ± standard deviation (n = 3). **p* < 0.05 versus untreated control cells.

Figure S3.

The siRNA-transfected cells were incubated for 48–72 h, followed by incubation with 400 ng mL^-1^ of mt or wt SubAB for 18 h. The *HRK* mRNA levels were measured using RT-qPCR. GAPDH served as the internal control. Data are presented as mean ± standard deviation (n = 3). **p* < 0.05.

Figure S4.

A, HeLa cells were incubated with 400 ng mL^-1^ of SubAB with or without 200 μM of RG108 or 5 mM of 5’-AZA for 18–24h. The *KLHDC7B* mRNA levels were measured using RT-qPCR. GAPDH served as the internal control. B, After the cells were treated using the aforementioned method, cell lysates underwent immunoblotting with antibodies. GAPDH served as the loading control. Densitometry was used to quantify the cPARP level in HeLa cells. Similar results were obtained from two independent experiments.
