# Supplementary figures and images for: A novel endoplasmic stress mediator, Kelch domain containing 7B (KLHDC7B), increased Harakiri (HRK) in the SubAB-induced apoptosis signaling pathway
Source: Cell Death Discov. 2021 Nov 19;7:360. doi: 10.1038/s41420-021-00753-0 (PMC8605022; doi:10.1038/s41420-021-00753-0)

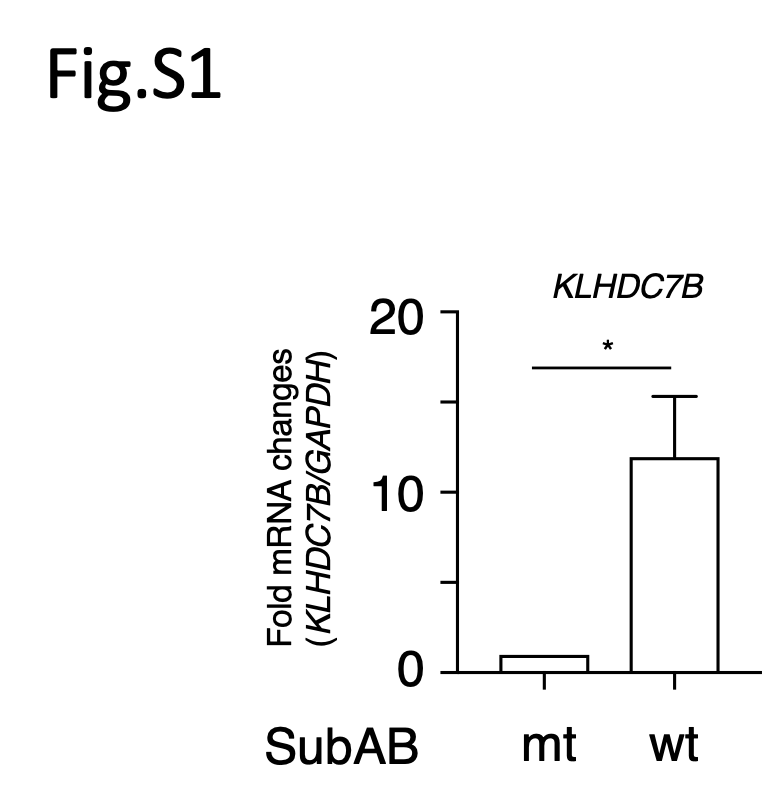

Supplement: Supplementary file 2 — Fig.S1 HCT116 cells were incubated with 400 ng mL of SubAB for 18 h. The KLHC7B mRNA levels were measured using RT-qPCR. GAPDH served as the internal control. Data are presented as mean± standard deviation (n = 3). *p < 0.05. [file 41420_2021_753_MOESM2_ESM.tif]

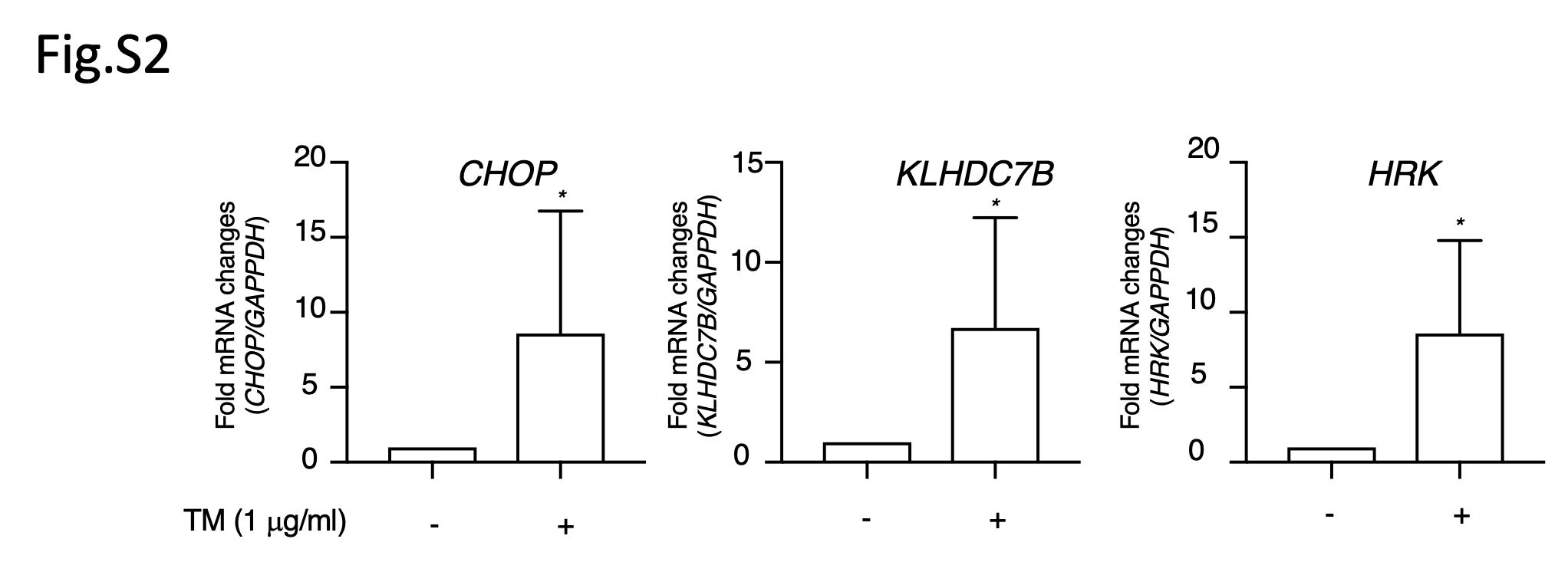

Supplement: Supplementary file 3 — Fig.S2 HeLa cells were incubated with 1 μg mL of Tunicamycin (TM) for 16 h. The KLHDC7B mRNA levels were measured using RT-qPCR. GAPDH served as the internal control. Data are presented as mean ± standard deviation (n = 3). *p < 0.05 versus untreated control cells. [file 41420_2021_753_MOESM3_ESM.tif]

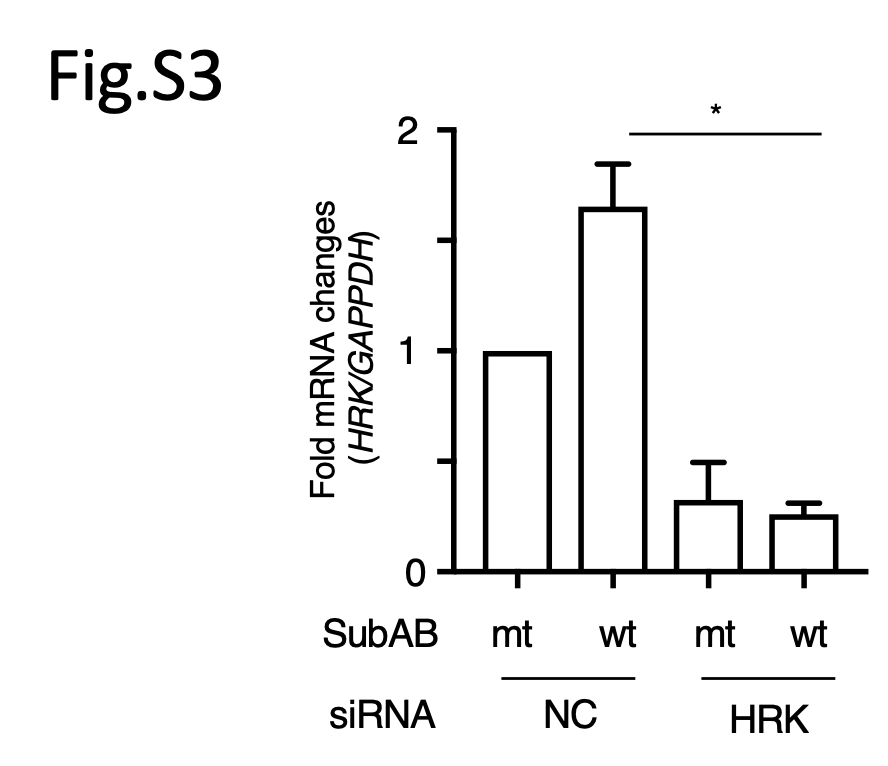

Supplement: Supplementary file 4 — Fig.S3 The siRNA-transfected cells were incubated for 48–72 h, followed by incubation with 400 ng mL of mt or wt SubAB for 18 h. The HRK mRNA levels were measured using RT-qPCR. GAPDH served as the internal control. Data are presented as mean ± standard deviation (n = 3). *p < 0.05. [file 41420_2021_753_MOESM4_ESM.tif]

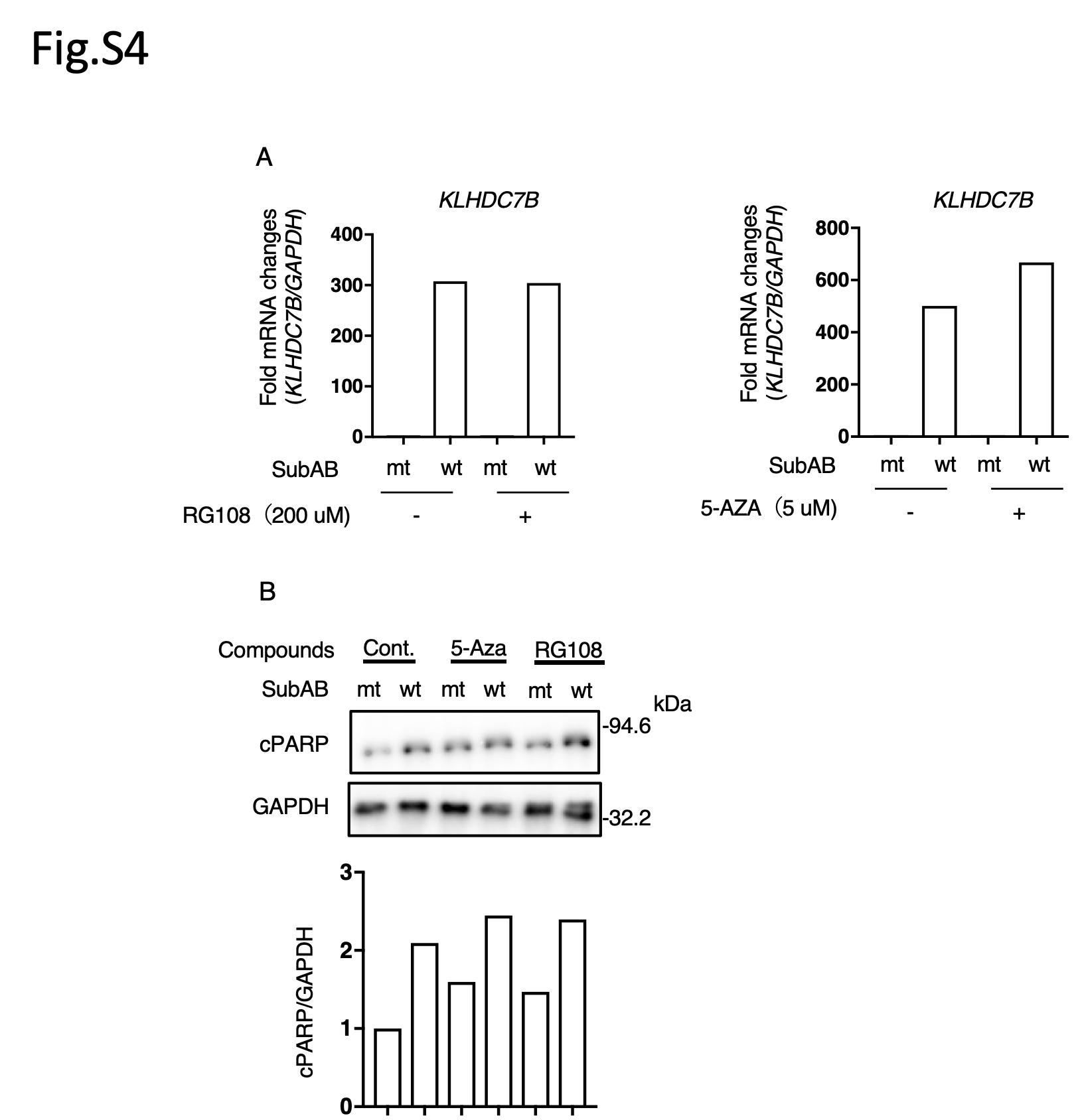

Supplement: Supplementary file 5 — Fig.S4 A, HeLa cells were incubated with 400 ng mL of SubAB with or without 200 μM of RG108 or 5 mM of 5’-AZA for 18–24h. The KLHDC7B mRNA levels were measured using RT-qPCR. GAPDH served as the internal control. B, After the cells were treated using the aforementioned method, cell lysates underwent immunoblotting with antibodies. GAPDH served as the loading control. Densitometry was used to quantify the cPARP level in HeLa cells. Similar results were obtained from two independent experiments. [file 41420_2021_753_MOESM5_ESM.tif]
